# GSDME-mediated pyroptosis promotes anti-tumor immunity of neoadjuvant chemotherapy in breast cancer

**DOI:** 10.1007/s00262-024-03752-z

**Published:** 2024-07-02

**Authors:** Changfang Fu, Wenbo Ji, Qianwen Cui, Anling Chen, Haiyan Weng, Nannan Lu, Wulin Yang

**Affiliations:** 1 Department of Pharmacy, The First Affiliated Hospital of USTC, Division of Life Sciences and Medicine, University of Science and Technology of China, Hefei, 230001 Anhui China; 2 Anhui Provincial Key Laboratory of Precision Pharmaceutical Preparations and Clinical Pharmacy, Hefei, 230001 Anhui China; 3 Clinical Pharmacy Department, Anhui Provincial Children’s Hospital, Hefei, 230000 Anhui China; 4 Anhui Province Key Laboratory of Medical Physics and Technology, Institute of Health and Medical Technology, Hefei Institutes of Physical Science, Chinese Academy of Sciences, Hefei, 230031 China; 5 Department of Pathology, The First Affiliated Hospital of USTC, Division of Life Sciences and Medicine, University of Science and Technology of China, Hefei, 230001 Anhui China; 6 Department of Oncology, The First Affiliated Hospital of USTC, Division of Life Sciences and Medicine, University of Science and Technology of China, Hefei, 230001 Anhui China; 7 Hefei Cancer Hospital, Chinese Academy of Sciences, Hefei, 230031 China

**Keywords:** Breast cancer, Neoadjuvant chemotherapy, Pathological complete response, Pyroptosis, Immunogenic cell death

## Abstract

Paclitaxel and anthracycline-based chemotherapy is one of the standard treatment options for breast cancer. However, only about 6–30% of breast cancer patients achieved a pathological complete response (pCR), and the mechanism responsible for the difference is still unclear. In this study, random forest algorithm was used to screen feature genes, and artificial neural network (ANN) algorithm was used to construct an ANN model for predicting the efficacy of neoadjuvant chemotherapy for breast cancer. Furthermore, digital pathology, cytology, and molecular biology experiments were used to verify the relationship between the efficacy of neoadjuvant chemotherapy and immune ecology. It was found that paclitaxel and doxorubicin, an anthracycline, could induce typical pyroptosis and bubbling in breast cancer cells, accompanied by gasdermin E (GSDME) cleavage. Paclitaxel with LDH release and Annexin V/PI doubule positive cell populations, and accompanied by the increased release of damage-associated molecular patterns, HMGB1 and ATP. Cell coculture experiments also demonstrated enhanced phagocytosis of macrophages and increased the levels of IFN-γ and IL-2 secretion after paclitaxel treatment. Mechanistically, GSDME may mediate paclitaxel and doxorubicin-induced pyroptosis in breast cancer cells through the caspase-9/caspase-3 pathway, activate anti-tumor immunity, and promote the efficacy of paclitaxel and anthracycline-based neoadjuvant chemotherapy. This study has practical guiding significance for the precision treatment of breast cancer, and can also provide ideas for understanding molecular mechanisms related to the chemotherapy sensitivity.

**Supplementary Information:**

The online version contains supplementary material available at 10.1007/s00262-024-03752-z.

## Introduction

Breast cancer is highly heterogeneous, different molecular types have different therapeutic responses and prognoses, and the same molecular type of breast cancer may also show different therapeutic sensitivity [1]. Chemotherapy is the cornerstone of systemic treatment of breast cancer [2]. Neoadjuvant chemotherapy based on paclitaxel and anthracycline is one of the standard treatment regimens for breast cancer. However, the pathological complete response (pCR) rate of this neoadjuvant chemotherapy is only 6–30% [3]. Meta-analyses of more than 100,000 breast cancer patients have shown that chemotherapy only reduces breast cancer recurrence and mortality by 20–33%, and only a small number of patients will benefit from chemotherapy, so many patients are overtreated [4]. At present, the reasons for the differences in sensitivity to neoadjuvant chemotherapy are not clear, and there is no effective method to predict the sensitivity.

Recent studies have shown that immune cells can drive important clinical features that affect breast cancer treatment outcomes, especially for the more aggressive proliferative subtypes of breast cancer, such as triple-negative breast cancer (TNBC) and HER2-positive breast cancer. Tumor stroma contains more tumor-infiltrating lymphocytes (TILs) of patients may have a better response to chemotherapy and prognosis [5–7]. In patients with TNBC and HER2-positive breast cancer, immune cell infiltration is detectable in up to 75% of patients and particularly dense infiltration in up to 20% of patients, while the number of TILs is low in the luminal subtypes [8, 9]. The pCR rate of neoadjuvant chemotherapy can reach 30–50% in patients with TNBC and HER2-positive breast cancer, while the pCR rate of neoadjuvant chemotherapy is only 5–15% in patients with luminal subtypes [8, 10].

In addition, the tumor microenvironment is often characterized by immunosuppression. The ultimate ability to drive the acquisition of anti-tumor immunity depends not only on the innate immune profile of the host but also on the initiating stimulus and dying cells [11, 12]. Recent studies have shown that some chemotherapy drugs can induce immunogenic cell death (ICD) to activate the immune system [11, 13]. For example, doxorubicin has been found to activate the caspase-3/gasdermin E (GSDME) pathway, triggering pyroptosis, which may affect the clinical outcome of cancer patients [14]. GSDME is the executor of pyroptosis [15–17]. Caspase-3 can cleave GSDME and convert apoptosis of GSDME-expressing cells into pyroptosis. Pyroptotic cells activate the immune system through the release of damage-associated molecular patterns (DAMPs) [18], which further triggers ICD and enhances the response to chemotherapy.

According to the gene expression of breast tumor tissue before neoadjuvant chemotherapy and the outcome of chemotherapy, we have developed 25 gene expression signatures to predict the efficacy of neoadjuvant chemotherapy for breast cancer [19]. Enrichment analysis found that 25 genes were mainly enriched in immune-related biological processes, indicating that the immune microenvironment may indeed mediate the sensitivity of neoadjuvant chemotherapy in breast cancer. To further optimize the model, random forest (RF) machine learning was used to screen the feature genes, and an artificial neural network (ANN) algorithm was used to construct an ANN model for predicting the efficacy of neoadjuvant chemotherapy based on paclitaxel and anthracycline for breast cancer. Furthermore, digital pathology, cytology, and molecular biology experiments were combined to investigate whether paclitaxel and anthracycline could induce pyroptosis of breast cancer cells and promote the release of DAMPs, thereby activating ICD and promoting the effect of neoadjuvant chemotherapy.

## Materials and methods

### Cell culture

The cell lines used in this study include human breast cancer cell lines MDA-MB-231, MCF-7, and T47D, mouse breast cancer cell lines EMT-6, and human monocytes THP-1 cell line. All cells were cultured at 37 °C with 5% CO_2_ in a medium supplemented with 10% Fetal Bovine Serum (FBS) (Lonsera, Uruguay) and 1% penicillin–streptomycin (PS) (SV30010, Hyclone). The EMT-6, T47D, and THP-1 cells used Roswell Park Memorial Institute (RPMI) 1640 medium. MDA-MB-231 and MCF-7 cells were cultured in the DMEM medium. Human peripheral blood mononuclear cells (PBMCs) were purchased from Hycells (Shanghai) with informed consent from the donors and cultured in RPMI 1640 medium.

For cells treated with drugs, the cells were generally incubated overnight until the cell density reached 60% and incubated for a while in a fresh medium containing the corresponding drugs. Unless otherwise specified, paclitaxel was used at a concentration of 10 nM and doxorubicin at a concentration of 10 μM.

### Antibodies and reagents

Antibody to GSDME (ab215191) was purchased from Abcam. Caspase-3 antibody (9662S), cleaved caspase-3 (9664S), caspase-9 antibody (9502S), and horseradish peroxidase-labeled rabbit secondary antibody (7074S) were purchased from Cell Signaling Technology (CST). β-acin (TA811000) was purchased from OriGene.

Paclitaxel (HY-B0015) and doxorubicin (HY-15142) from MedChemExpress. ATP assay kits (ATPLite, 6016736) were purchased from PerkinElmer. Human high-mobility group protein B1 (HMGB1) enzyme-linked immunosorbent assay (ELISA) kit (E-EL-H1554c) was purchased from Elabscience. The cytotoxicity assay kit (G1780) was purchased from Promega. Annexin V-FITC/PI apoptosis detection kit (556547) and antihuman CD14 Magnetic Particles-DM (23210402) were ordered from BD Biosciences company. EasySep™ Human T CellIsolation Kit (1000149980) and Interleukin (IL)-6 (78050.1-20UG) were ordered from Stemcell. GM-CSF (300-03-100UG), IL-4 (200-04-5UG), IL-1β (200-0113-10UG) and TNF-α (300-01A-10UG) were obtained from Peprotech. PGE2 (P860711-5MG) was purchased from Macklin.

### Gene sets collection and processing

Microarray expression datasets and platform files for breast cancer patients receiving neoadjuvant chemotherapy with paclitaxel and anthracycline were downloaded from the GEO database (http://www.ncbi.nlm.nih.gov/geo/) [20]. Neoadjuvant chemotherapy regimens included patients treated with paclitaxel followed by fluorouracil, anthracycline, and cyclophosphamide (T/FAC) regimen or paclitaxel followed by anthracycline and cyclophosphamide (T/AC) regimen. After extracting gene expression data and excluding all samples with incomplete data, a total of 744 patients from GSE32646 [21], GSE20271 [22], GSE20194 [23, 24], GSE25055 [25], and GSE41998 [26] were included in this study (Supplemental Table 1). The R package “affyPLM” was used to perform background correction, quantile normalization, and log_2_ transformation of the raw data using the RMA algorithm [27].

**Table 1:** Clinical characteristics of patients

Characteristic	pCR (*n* = 10)	RD (*n* = 26)
Age (years)		
< 65	10	24
≥ 65	0	2
Tumor stage		
T0	0	0
T1	1	2
T2	8	20
T3	1	4
T4	0	0
Lymph node status		
Positive	6	15
Negative	3	7
NA	1	4
Histological grade		
1	0	0
2	2	9
3	0	6
NA	8	11
ER status		
Positive	3	17
Negative	7	9
PR status		
Positive	0	12
Negative	10	14
HER2 status		
Positive	5	5
Negative	5	21
Neoadjuvant therapy		
TAC	8	21
T/AC	1	4
TH/AC	1	1

*T* paclitaxel, *A* anthracycline, *C* cyclophosphamide, *H* herceptin

### Patient clinical data

In this study, breast biopsy tissue samples before neoadjuvant therapy and breast surgery tissue samples after neoadjuvant therapy and corresponding clinicopathological data of breast cancer patients were retrospectively collected and analyzed. The study was approved by the Ethics Committee of the First Affiliated Hospital of USTC, Department of Life Sciences and Medicine, USTC (No. 2022KY286). A total of 36 breast cancer patients with stage I to III who were treated in the First Affiliated Hospital of USTC from September 2019 to April 2021 were included (Table 1). All patients were pathologically confirmed to have invasive breast cancer and received 4–6 cycles of neoadjuvant chemotherapy with T/FAC or T/AC regimens. Pregnant or lactating women and patients with serious comorbidities were excluded.

### Pathological evaluation of response to neoadjuvant chemotherapy

After the neoadjuvant chemotherapy course, all patients underwent modified radical mastectomy or lumpectomy. The Miller-Payne (MP) grading system was used to evaluate the pathological response to neoadjuvant chemotherapy based on histological examination of surgical tissue specimens of breast cancer [28]. Number of cancer cells, on the whole, there is no change or only a small number of cancer cells to change for grade 1 (G1); the number of cancer cells reduced by less than 30% was defined as grade 2 (G2). The reduction of invasive cancer cells between 30 and 90% was grade 3 (G3); the number of cancer cells decreased by more than 90%, and only small clusters or single cancer cells remained, which was grade 4 (G4). The absence of invasive cancer cells was rated as grade 5 (G5). pCR after neoadjuvant chemotherapy was defined as the absence of invasive cancer (G5) and residual disease (RD) as the presence of residual invasive cancer cells in the breast and lymph nodes (G1-4). Pathological evaluation was performed collectively by two experienced breast pathologists.

### The evaluation of TILs

One HE-stained section (4–5 μm) was prepared from the breast biopsy of each breast cancer patient before neoadjuvant therapy. HE-stained sections were scanned using a whole slide imaging (WIS) system and converted to digital pathological sections [29–31]. The percentage of TILs in the breast tumor stroma was assessed using digital pathological slides (200–400 × magnification) according to the criteria established by the International Working Group on TILs [32]. TILs were finally reported as the percentage of stromal TILs in breast cancer tissue. TILs outside tumor borders and normal lobules were excluded. When the percentage of TILs is in doubt, the case can be discussed jointly with a second pathologist. TILs on digital pathology slides were fully evaluated in 10 regions, and the average TILs for each patient were finally reported.

According to the infiltration of TILs, the immune classification was divided into three types: immune-infiltrated phenotypes, immune-excluded phenotypes, and immune-desert phenotypes [33, 34]. The immune-infiltrated phenotypes had abundant TILs, and TILs could infiltrate into the tumor nests. The immune-excluded phenotypes also had a high level of TILs, but TILs were concentrated in the periphery of the tumor nest. However, there was little infiltration of TILs in the immune-desert phenotype. On this basis, the correlation between pathological MP grade and patients' age, T stage, molecular subtype, ER, PR, HER2 status, TIL content, and immunophenotype was analyzed.

### RF algorithm screens feature genes

GSE32646 and GSE20271 were merged into a training set and batch effects were removed by R package “sva.” The “limma” package [35] in R software was used to analyze the differentially expressed genes between pCR and RD samples in the training set, and the threshold was set as adjusted *p* < 0.05 and |log_2_ FC|> 0.5. The R package “pheatmap” was used to make heat maps, and then GSE201194, GSE25055, and GSE41998 were used as validation sets for verification.

To assess the importance of differentially expressed genes in pCR and RD samples, we used RF algorithm to screen feature genes through R package “random forest” [36, 37]. The number of trees with the lowest error rate and best stability was taken as the best mtry, and the Gini coefficient was used to measure the contribution of each gene to the RF model. The top 30 feature genes with the greatest decline in Gini index were selected and the enrichment analysis of gene ontology (GO) and Kyoto Encyclopedia of Genes and Genomes (KEGG) pathway was performed using the R software package “ClusterProfiler.” The *P* values of GO and KEGG enrichment analysis were set as 0.05 and 0.15, respectively.

### ANN model construction

Next, the ANN model was constructed based on the key genes screened by RF. Firstly, the key gene expression data in the training set was extracted and the gene score table was constructed. By calculating the median expression value of each gene, the gene was assigned according to the log_2_FC value, when log_2_ FC > 0, if the expression value of the gene was greater than the median expression value, the value is 1; Otherwise, it will be assigned the value 0. When log_2_FC < 0, if the gene expression value was greater than the median gene expression value, 0 will be assigned. Otherwise, assign a value of 1 [38, 39]. Therefore, the gene expression data from different platforms or different batches was constructed as a matrix of 0 s and 1 s, eliminating batch effects. The response to neoadjuvant chemotherapy was then used as the outcome variable, with pCR assigned a value of 1 and RD assigned a value of 0. Based on the above matrix, an ANN model was constructed with an R package “neuralnet,” five hidden layers were set as model parameters, and the weights of key genes were calculated by the ANN algorithm. GSE201194, GSE25055, and GSE41998 were used as validation sets to test the classification efficiency of the ANN model. The area under the receiver operating characteristic (ROC) curve (AUC) and 95% confidence interval (CI) were calculated using R packet “pROC” to evaluate the performance of the ANN model [40]. The calculation formula is as follows:

$$\text{Neural\, Score}=\sum \left(\text{GeneExpression}\times \text{NeuralNetworkWeight}\right).$$

### Optical microscope imaging

The cells were inoculated in 6-, 12-, or 24-well plates. In blind experiments, Olympus CKX53 was used to capture static bright-field images of pyroptosis cells (blown bubbles) at room temperature with TCapture software.

### Flow cytometry

After the cells were treated with the drug for 72 h, the cells were collected and washed with PBS. The cells were resuspended in the binding buffer and Annexin V-PITC/PI staining was performed with the Annexin V-FITC/PI apoptosis detection kit. Flow cytometry was performed according to kit instructions and the data were processed with FlowJo software.

### Lactate dehydrogenase (LDH) release assay

The cells were inoculated on 24-well plates and treated with 10 nM paclitaxel. The culture supernatant was collected and centrifuged at 1000 rpm for 10 min. Collect the supernatant and transfer it to a 96-well plate for detection according to CytoTox 96 test kit requirements. The formula for calculating LDH release percentage is (LDH_sample_-LDH_background_)/(LDH_maximum_-LDH_background_) × 100%. LDH_sample_, LDH_background_ and LDH_maximum_ are the OD490 values measured for the supernatant of the treated drug, the untreated drug, and the lysate (provided by the kit), respectively. Each sample was repeated three times to obtain an average value.

### HMGB1 and ATP assays

Cells were inoculated on 24-well plates, cultured to a density of 2 × 10^5^ cells/well, and treated with paclitaxel. The supernatant was collected and the HMGB1 level in the cell culture supernatant was detected by the HMGB1 ELISA kit. ATP level in cell culture supernatant were detected with an ATP detection kit. The Interleukin 1 Beta (IL-1β) level in cell culture supernatant were detected with an IL-1β ELISA kit [41].

### Western blot

Cultured cells were collected and lysed using RIPA buffer containing PMSF (1 mM). Total protein concentration was determined using the BCA Protein Assay kit (P0011, Beyotime), and cell protein extracts was separated by SDS-PAGE, transferred to PVDF membranes, and blocked with 5% nonfat milk powder, followed by incubation of membranes with primary antibodies overnight at 4 °C and with secondary antibodies for 1 h. All proteins were detected by Tanon High-sig ECL Western Blotting.

### Cell coculture experiment

THP-1 cells were treated with 200 ng/ml PMA (Sigma, P8139) for 6 h and then cultured in RPMI medium for 6 h to differentiate into M0 macrophages. M0 macrophages were polarized into M1 macrophages by treatment with 20 ng/ml (IFN)-γ (R&D, #285-IF) and 100 ng/ml LPS (Sigma, #8630). After that, M1 macrophages were cultured in RPMI 1640 for another 24 h [42].

MDA-MB-231 cells were pre-treated with paclitaxel to exclude the paclitaxel effect on THP-1 cells. MDA-MB-231 cells were cultured in RPMI 1640 medium free of serum and penicillin–streptomycin and stained with Hoechst 33342. M1 macrophages were cultured in serum-free and penicillin–streptomycin medium and stained with DiR iodide. MDA-MB-231 and M1 macrophages were cocultured in serum-free and penicillin–streptomycin medium for 30 min. The morphological changes of MDA-MB-231 and M1 macrophages after coculture were observed under an inverted microscope and the phagocytosis of MDA-MB-231 cells by M1 macrophages was counted.

### T cell activation assay

CD14+ mononuclear cells were purified from PBMCs (013K111) with antihuman CD14 magnetic particles-DM. Immature dendritic cells (DCs) were generated in RPMI 1640 with 1000 U/ml GM-CSF and 500 U/ml IL-4 for 5 days, and mature DCs were generated for another 2 days with 10 ng/ml IL-6, 10 mg/ml IL-1β, 10 ng/ml TNF-α and 1 µg/ml PGE2. Human primary CD3+ T cells were isolated from PBMCs (013K023) using EasySep™ Human T CellIsolation Kit, and then cocultured with mature DCs (1 × 10^4^ cells) at a ratio of 10:1 and MDA-MB-231 cells (1 × 10^4^ cells) in 96-well plates for 24 h. The levels of IFN-γ and IL-2 in the supernatant were detected by ELISA.

### Establishing stable GSDME-overexpressing cells

Lentivirus-mediated overexpression of GSDME against cells were purchased from SBI (HG-VMS1529). 4T1 cells were incubated in medium containing optimal dilutions of lentivirus mixed with polybrene. After 48 h of transfection, cells were subjected to puromycin selection (5 mg/mL) to obtain stably transfected GSDME-overexpressing 4T1 (4T1-GSDME) cells.

### Statistics

All data were processed using R (Version 3.6.1, https://www.R-project.org) software. Differential gene analysis between pCR and RD samples was performed using the R package “limma.” RF algorithm was used to screen the feature genes, and the ANN algorithm was used to construct the prediction model. The Wilcoxon test was used for differences between two groups, and the Kruskal–Wallis test was used for differences between three or more groups. Correlations between variables were analyzed using the Pearson method. Data were expressed as Mean ± Standard Deviation (SD). *p* < 0.05 indicates the difference was statistically significant. *is represented as *p* < 0.05, **is represented as *p* < 0.01.

## Results

### The signature genes of chemotherapy sensitivity were screened by RF

**Fig. 1:**
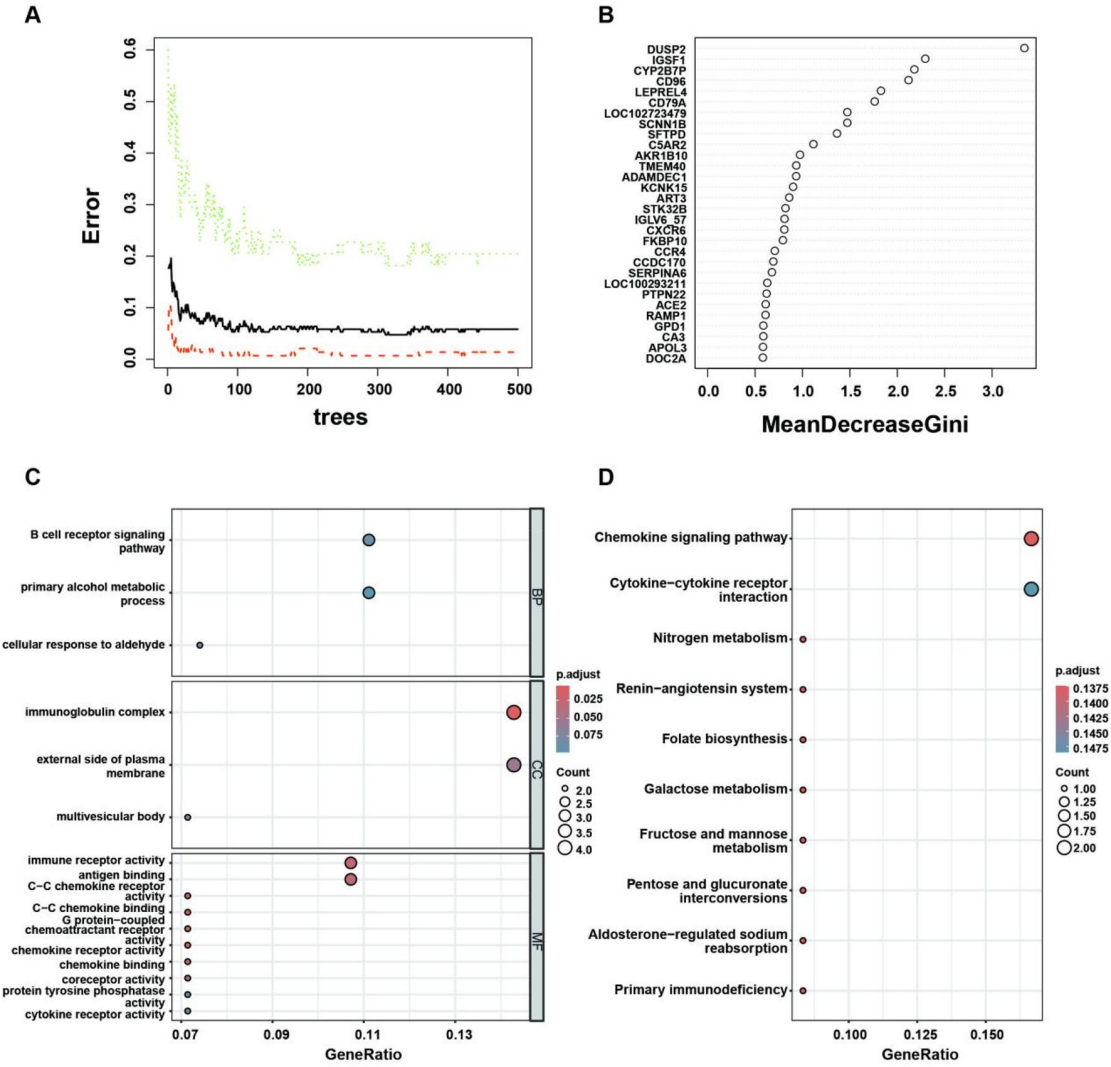
RF screening feature genes. **A** The influence of the number of decision trees on the error rate. The X-axis represents the number of decision trees and the Y-axis represents the error rate. When the number of decision trees is about 311, the error rate is relatively stable. **B** The Gini coefficient method in RF classifier to select important genes. Grouping by pCR and RD, the input variables of the RF were sorted to screen 30 significant genes. The X-axis is the importance index and the Y-axis is the gene variable. **C** GO enrichment analysis of feature genes. **D** KEGG pathways significantly associated with the feature genes screened by RF

The GEO database of breast cancer patients receiving neoadjuvant chemotherapy was collected. Using *p* < 0.05, |log_2_FC|> 0.5 as screening criteria, a total of 351 differential genes were identified between pCR samples and RD samples in the training set. As shown in the heat map, the expression matrix of 351 differential genes was clearly distinguished between pCR and RD samples (Supplemental Fig. 1A). The feature genes were then screened from 351 differential genes using the RF algorithm. To find the optimal parameter, we classify all possible genetic variables by RF cycle and calculate the average error rate of the model. As shown in Fig. 1A, the RF model has the lowest out-of-band error rate when the optimal number of variables for binary trees is 4 and the optimal number of stable trees is 311. The RF model is constructed using the Gini coefficient method, that is, the method of reducing precision and mean square error, to measure the importance of the output variables. Finally, 4 variables and 311 trees were selected as the final parameters of the RF classifier to obtain the dimensional importance of all variables. MeanDecreaseAccuracy and MeanDecreaseGini of the top 30 important genes are shown in Fig. 1B.

Then, the enrichment analysis of GO and KEGG pathway was performed on the top 30 important genes during the Gini coefficient decline. The significant enrichment of GO in biological processes (CC) includes the “immunoglobulin complex” (Fig. 1C). The significant enrichment of GO in molecular function (MF) includes “immune receptor activity” “antigen binding” (Fig. 1C). KEGG enrichment results showed that these genes were mainly related to the “chemokine signaling pathway” (Fig. 1D). These results indicate that signature genes obtained by RF screening are mainly involved in immune response processes/pathways, suggesting that immune regulation may be involved in sensitivity regulation of neoadjuvant chemotherapy for breast cancer.

### ANN model construction for predicting neoadjuvant chemotherapy sensitivity in breast cancer

**Fig. 2:**
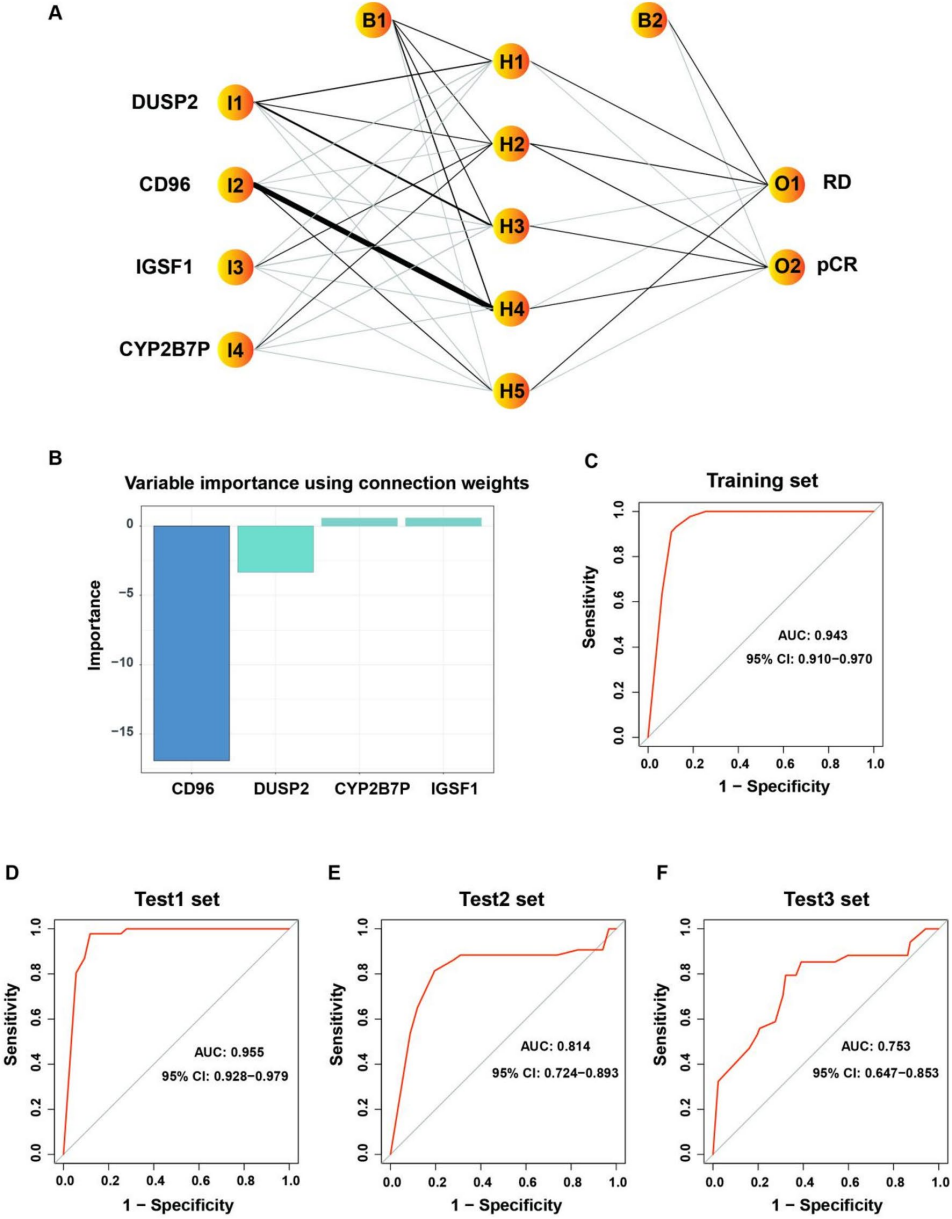
ANN model construction. **A** Results of the ANN visualization. Labels outside the node denote variable names, and labels inside the node denote layers and nodes (I: input, H: hidden, O: output, B: bias). **B** Variable importance with Olden’s algorithm. **C–F** AUC of training and test sets. Training set, GSE32646 and GSE20271. Test1 set, GSE20194. Test2 set, GSE25055. Test3 set, GSE41998

Among the top 30 differential genes, there are 4 MeanDecreaseGini > 2 genes, DUSP2, IGSF1, CYP2B7P, and CD96 (Fig. 1B). Based on the above 4 MeanDecreaseGini > 2 genes to construct ANN prediction model. The ANN model has 4 input layers, 5 hidden layers, and 2 output layers (Fig. 2A). A total of 845 steps were taken during the entire training process. When the termination condition reaches the threshold (< 0.01), the absolute partial derivative of the error function is 0.009597032. Olden's algorithm shows the weights of the four key genes (Fig. 2B). As shown in Fig. 2C, the AUC value of the training set is 0.943 (95% CI 0.910–0.970), indicating that the model has good predictive performance. The ANN classifier was further validated in two independent T/FAC-based test sets GSE20194 and GSE25055 and one T/AC-based test set GSE41998, yielding AUC values of 0.955 (95% CI 0.928–0.979) (Fig. 2D), 0.814 (95% CI 0.724–0.893) (Fig. 2E) and 0.753 (95% CI 0.647–0.853) (Fig. 2F), respectively, further validating the good performance of the ANN model.

Among the four key genes in the ANN model, CD96 had the largest weight and was the common gene with a previously constructed 25-feature-gene model (Supplemental Fig. 1B). Further analysis using PanglaoDB database showed that CD96 was mainly expressed in T cells, NK cells, Gamma delta T cells, etc. They were predominantly in T cells (Supplemental Fig. 1C). These results also suggest that immune cells play an essential role in the efficacy of neoadjuvant chemotherapy in breast cancer. In addition to directly killing tumor cells, how to interact with immune cells is the key for chemotherapeutic drugs to anti-tumor.

### The TILs in breast tumor tissue is correlated with the efficacy of neoadjuvant chemotherapy

**Fig. 3:**
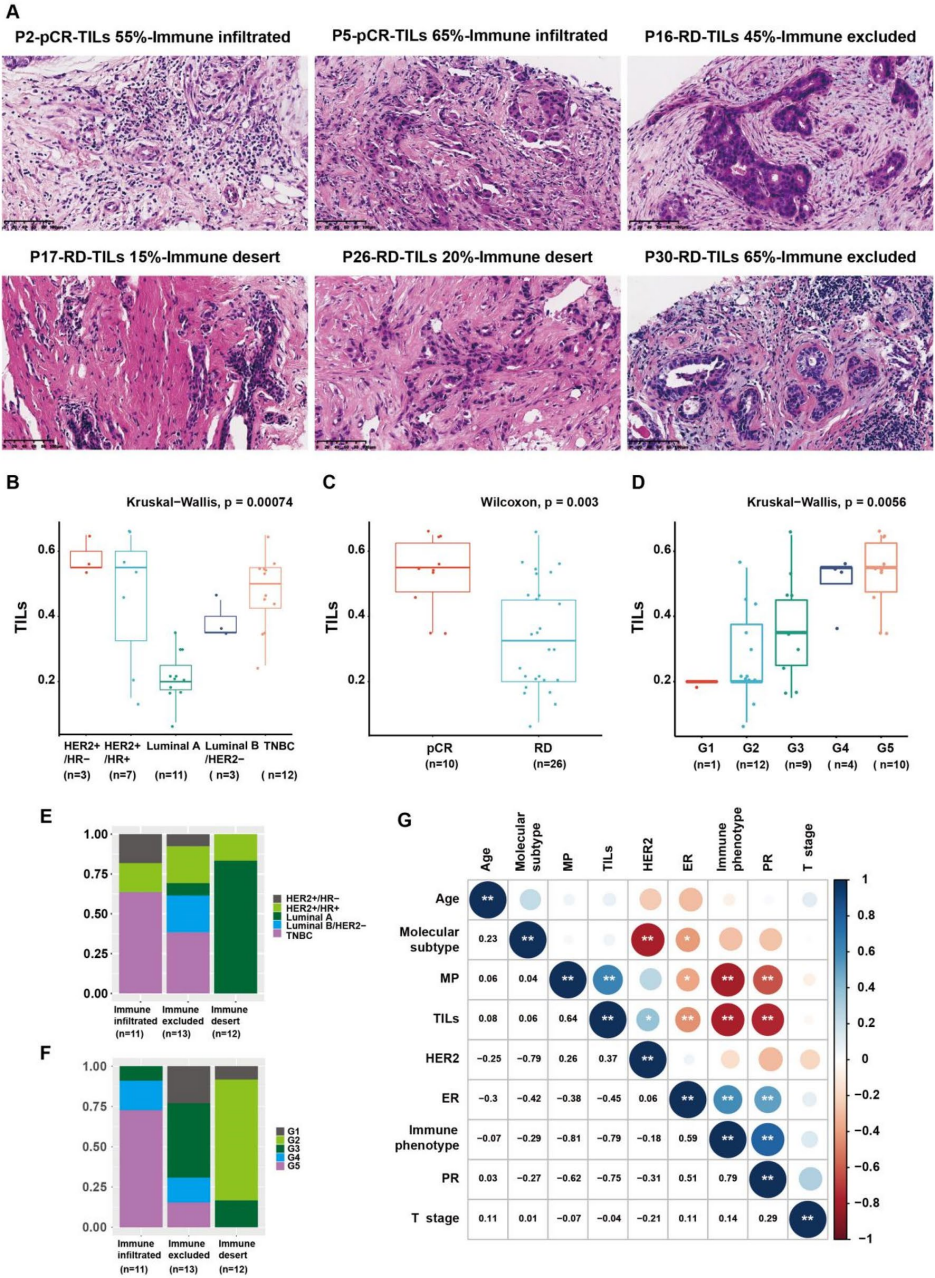
Breast tumor tissue TILs related to the curative effect of chemotherapy. **A** Representative digital pathological slides showing TIL content and immunophenotyping of breast tumor tissue. **B** The content of TILs in breast tumor tissues of TNBC and HER2+ breast cancer before treatment was significantly higher than that of luminal breast cancer. **C** The content of TILs in breast tumor tissues of pCR patients before treatment was significantly higher than that of RD patients. **D** The higher the MP grade of neoadjuvant chemotherapy, the higher the TIL content of breast tumor tissue before treatment. **E** TNBC and HER2+ breast cancer were mainly immune infiltrated, while luminal breast cancer was mainly immune-desert phenotype and immune-excluded phenotype. **F** pCR (MP grade G5), mainly immune-infiltrated phenotype, RD (MP grade G1-4), mainly immune-excluded phenotype and immune-desert phenotype. **G** The MP grade was highly positively correlated with the content of TILs, that is, the higher the content of TILs, the higher the MP grade

Since enrichment analysis showed that the efficacy of neoadjuvant chemotherapy in breast cancer may be related to the immune process, we evaluated the relationship between the efficacy of neoadjuvant chemotherapy with paclitaxel and anthracycline and the content of TILs and immunophenotypes in breast tumor tissues based on digital pathological slides. The TILs in breast tumor tissue include interstitial TILs and intratumoral TILs. Most TILs are located in the stroma, and only a few TILs come into direct contact with tumor cells within the tumor. Intratumoral TILs are difficult to observe on biopsy, so interstitial TILs are often used as the main parameter for the abundance of lymphocyte infiltration in breast tumor tissue. Ten areas were randomly selected in digital pathology slides, and the average of the percentage of TILs infiltrated was calculated to represent the TIL content for each patient. See the Methods section for details. According to the TILs evaluation criteria established by the International TILs Working Group, based on digital pathological slides (Fig. 3A, Supplemental Fig. 2, Supplemental Fig. 3), it was seen that the number of TILs in TNBC and HER2+ subtypes was higher than that in luminal subtypes (Fig. 3B). The amount of TILs in breast tumor tissue before treatment was significantly higher in pCR samples than in RD samples (Fig. 3C). Among them, MP grade G5 (pCR) patients had the highest TIL content. As the MP grade decreased, the TILs content decreased (Fig. 3D).

In addition, the immunophenotyping results of breast tumor tissues before treatment showed that TNBC and HER2+ breast cancer were mainly immune-infiltrated phenotype, and luminal breast cancer was mainly immune-desert phenotype and immune-excluded phenotype (Fig. 3E). The pCR samples with MP grade G5 were mainly immune infiltrated, and the pCR samples with MP grade G1-4 were mainly immune excluded and immune desert (Fig. 3F), indicating that immune-infiltrating breast cancer patients were more likely to obtain pCR after neoadjuvant chemotherapy. Further Pearson correlation analysis showed that MP grading was highly positively correlated with TIL content, that is, the higher the TILs content, the higher the MP grading. The high MP grade of the immune-infiltrated phenotype and the low MP grade of the immune-desert phenotype also suggested that the MP grade of breast cancer after neoadjuvant chemotherapy was mainly related to the immune status of breast tumor tissue before treatment (Fig. 3G). These results indicate that the immune status of breast tumor tissues before treatment is highly correlated with the efficacy of neoadjuvant chemotherapy, suggesting that the immune ecosystem may be involved in regulating the sensitivity of neoadjuvant chemotherapy in breast cancer.

### GSDME mediates paclitaxel-induced pyroptosis through the caspase-9/caspase-3 pathway

**Fig. 4:**
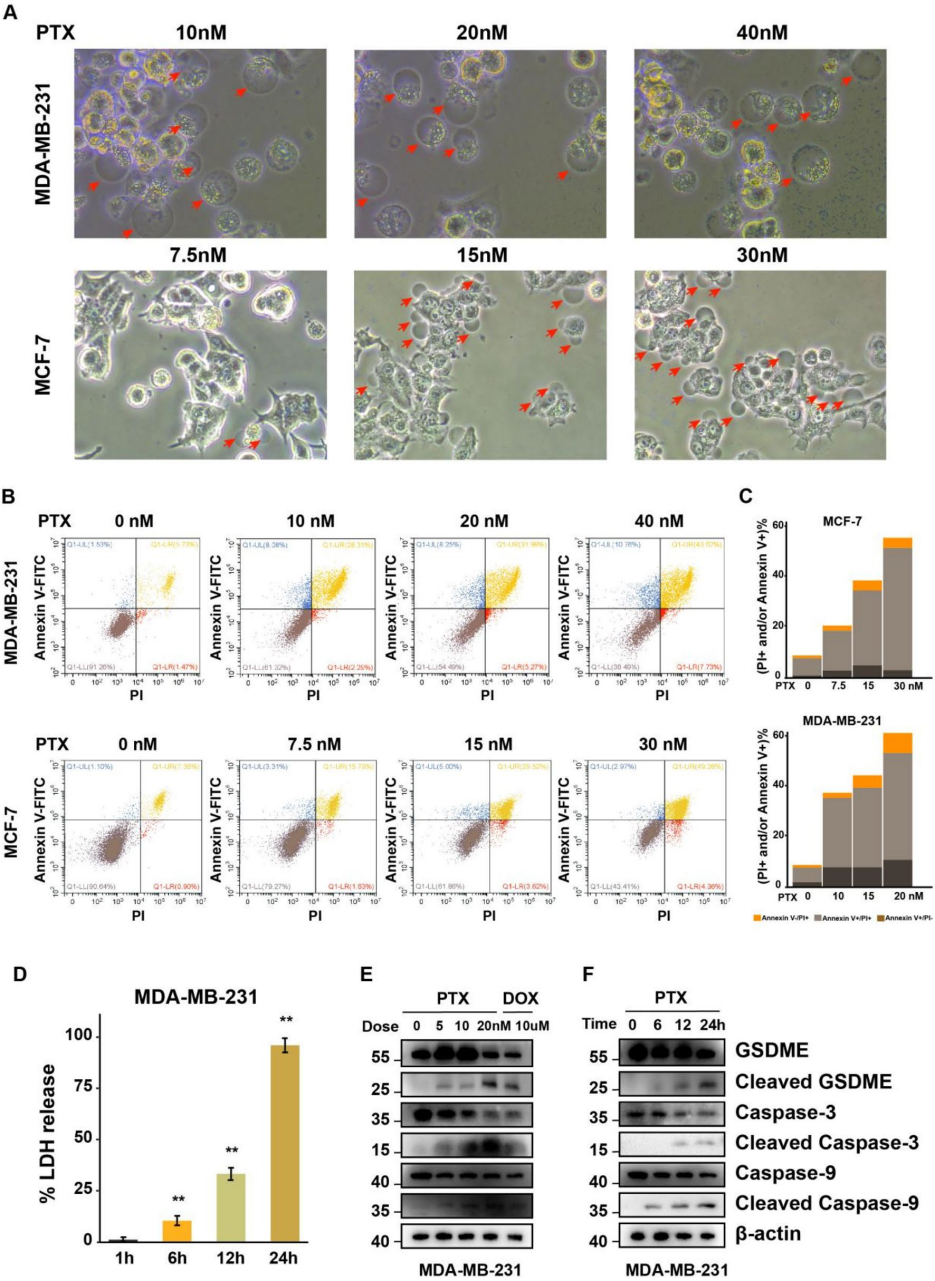
Paclitaxel induces pyroptosis in breast cancer cells. **A** Typical morphological features of pyroptosis in MDA-MB-231 and MCF-7 cells with high GSDME expression after paclitaxel administration. **B**, **C** Flow cytometric analysis of cell death with Annexin V/PI staining (**B**) and quantification (**C**). **D** Increased LDH release in MDA-MB-231 cells at 24 h after paclitaxel administration (10 nM). **E** MDA-MB-231 cells with high GSDME expression showed a dose-dependent increase in pyroptosis-related proteins after treatment with a range dose of paclitaxel (0–20 nM) for 24 h. **F** MDA-MB-231 cells with high GSDME expression showed a time-dependent increase in pyroptosis-related proteins after paclitaxel administration (10 nM). **p < 0.01

Recent studies have shown that some chemotherapy drugs can induce ICD. Pyroptosis is a type of ICD. It has long been reported that doxorubicin can induce cell pyroptosis. GSDME is the executor of chemotherapy-induced pyroptosis. In this study, high expression of GSDME protein was detected in breast cancer MDA-MB-231 and MCF-7 cells, but not in T47D and EMT-6 cells (Supplemental Fig. 4A). After doxorubicin administration, breast cancer MDA-MB-231 and MCF-7 cells, but not EMT-6 cells, showed typical morphological features of pyroptosis (Supplemental Fig. 4B). Meanwhile, after doxorubicin treatment, MDA-MB-231 cells with high GSDME expression showed GSDME cleavage (Fig. 4E). These results indicate that doxorubicin induces pyroptosis in breast cancer cells with high GSDME expression. Furthermore, we investigated whether paclitaxel could induce pyroptosis in breast cancer cells. After paclitaxel administration, typical morphological features of pyroptosis, including cell swelling and bullae formation at the plasma membrane, were also observed in MDA-MB-231 and MCF-7 cells with high GSDME expression (Fig. 4A), but not in T47D and EMT-6 cells with low GSDME expression (Supplemental Fig. 4C). It has been reported that GSDME-mediated pyroptosis can form membrane pores, leading to loss of integrity of the cell membrane. We also observed Annexin V+/PI+ cells (Fig. 4B and C) and increased LDH release (Fig. 4D) in MDA-MB-231 cells. Paclitaxel-treated MDA-MB-231 cells showed GSDME cleavage (Fig. 4E), suggesting that paclitaxel may induce pyroptosis in breast cancer cells with high GSDME expression.

In another cell line 4T1, the same phenomenon was seen that paclitaxel could induce pyroptosis in GSDME-overexpressing 4T1 cells but had no effect on wild-type 4T1 cells. After paclitaxel treatment (50 nM), the cells overexpressing GSDME showed typical morphological features related to pyroptosis (Supplemental Fig. 5A and B). The level of IL-1β in the cell culture supernatant was also significantly increased after treatment with paclitaxel (50 nM) (Supplemental Fig. 5C).

**Fig. 5:**
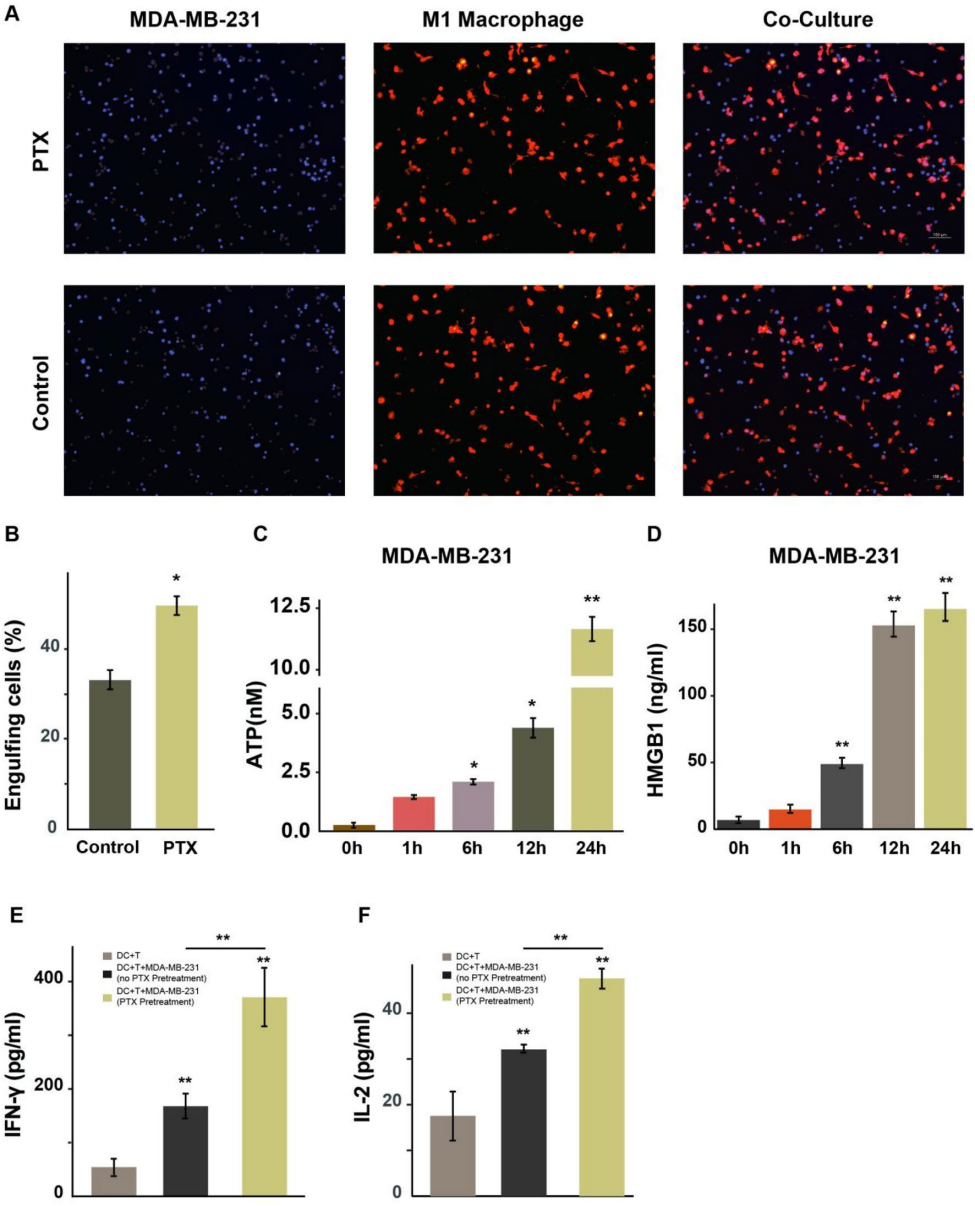
Cell coculture experiment. **A** Representative images of MDA-MB-231 cells cocultured with M1 macrophages, scale bars represent 100 µm. **B** Quantification of macrophages engulfing breast cancer cells. (C&D) The levels of ATP (**C**) and HMGB1 (**D**) in the supernatant after paclitaxel treatment (10 nM) were determined by chemiluminescence and enzyme-linked immunosorbent assay, respectively. **E, F** PTX-treated MDA-MB-231 cells cocultured with mature DCs and CD3+ T cells for 24 h, and then the levels of IFN-γ (**E**) and IL-2 (**F**) in the supernatant were detected by ELISA, respectively. DCs, dendritic cells. *p < 0.05; **p < 0.01

Caspase-3 has been reported to cleave GSDME upon activation to trigger pyroptosis. MDA-MB-231 cells were treated with a range dose of paclitaxel (0–20 nM) for 24 h and with a 10 nM paclitaxel at different time (0–24 h), respectively, we found an increase in GSDME cleavage and caspase-3 activation in MDA-MB-231 cells with high GSDME expression. We further examined the activation of caspase-9 protein in the mitochondrial-mediated death pathway and found that cleaved caspase-9 protein was significantly increased in MDA-MB-231 cells after paclitaxel treatment (Fig. 4E and F). These results indicated that GSDME cleavage and activation of caspase-9 and caspase-3 proteins occurred in breast cancer cells with high GSDME expression after paclitaxel treatment, suggesting that GSDME may mediate paclitaxel-induced pyroptosis in breast cancer cells through the caspase-9/caspase-3 pathway.

### GSDME-mediated pyroptosis promotes anti-tumor immunity

To further clarify the relationship between paclitaxel-induced pyroptosis of breast cancer cells and tumor immune microenvironment, we performed cell coculture experiments. Human monocyte line THP-1 cells were selected and induced by classical PMA to simulate the differentiation of monocytes into macrophages in vitro. First, THP-1 cells were induced into M0 macrophages with PMA and then stimulated with different concentrations of LPS combined with IFN-γ to generate M1 cells. Finally, MDA-MB-231 and M1 macrophages were cocultured for 1 h, and the number of MDA-MB-231 engulfed by M1 macrophages was counted (Fig. 5A and B). As a result, the number of breast cancer MDA-MB-231 cells phagocytosed by M1 macrophages was increased in the paclitaxel-treated group as compared with the control group that did not receive paclitaxel (Fig. 5B). In addition, as shown in Fig. 5C and D, the release of two DAMPs, HMGB1 and ATP, increased in a time-dependent manner in breast cancer cells treated with paclitaxel. To assess the ability of GSDME-mediated pyroptosis to promote anti-tumor immunity of neoadjuvant chemotherapy in breast cancer, paclitaxel-treated MDA-MB-231 cells with high GSDME expression were cocultured with mature DCs and CD3+ T cells for 24 h, and then IFN-γand IL-2 secretion levels in the supernatant were detected by ELISA, respectively. The results showed that paclitaxel-treated MDA-MB-231 cells induced CD3+ T cells activation, as measured by an increase in IFN-γ (Fig. 5E) and IL-2 (Fig. 5F) secretion at 24 h. These results suggest that paclitaxel may induce pyroptosis in breast cancer cells with high GSDME expression and enhance anti-tumor immunity by releasing DAMPs to promote ICD regulated by macrophages.

## Discussion

In this study, RF algorithm was used to screen feature genes and ANN algorithm was used to construct a prediction model for breast cancer response to neoadjuvant chemotherapy. The ROC curve showed that the prediction performance of the model was good. Gene enrichment analysis combined with pathological and cytological experiments suggested that the immune ecosystem may be involved in regulating the sensitivity of neoadjuvant chemotherapy based on paclitaxel and anthracycline in breast cancer, and this process may be related to pyroptosis-induced ICD. To understand how the immune ecosystem enhances the sensitization of chemotherapeutic drugs to tumor cells, we proposed the hypothesis that paclitaxel-induced tumor cell pyroptosis could promote anti-tumor immunity, which was verified in subsequent experiments.

It has been reported that immune cells can drive important clinical features that affect the treatment outcome of breast cancer [8, 43]. Patients with high TILs in breast tumor stroma may have better response and prognosis to chemotherapy [5]. To test the pathological link between the immune ecosystem and chemotherapy sensitivity, we evaluated digital pathological slides of breast tumor tissues from 36 patients who received neoadjuvant chemotherapy with paclitaxel and anthracycline in our hospital. We found that TILs was higher in breast tumor tissues of patients with pCR before treatment, which was mainly immune-infiltrated phenotype, and lower in patients with RD. They were mainly immune-desert phenotype and immune-excluded phenotype. There is considerable evidence that the immune system plays a crucial role in the clinical outcome of some molecular subtypes of breast cancer, especially the more aggressive subtypes, such as TNBC and HER2+ breast cancer [7, 12]. The present study found that TNBC and HER2+ breast cancer patients have a high abundance of TILs infiltration, mainly immune infiltrated, while luminal breast cancer patients have a low abundance of TILs infiltration, mainly immune desert and immune excluded, further suggesting that the tumor immune microenvironment of breast cancer patients may affect the efficacy of neoadjuvant chemotherapy.

However, the tumor tissue microenvironment often exhibits immunosuppressive features that may prevent the initiation or execution of anti-tumor immunity [9, 44]. The ultimate ability to drive anti-tumor-acquired immunity depends not only on the innate immune profile of the host but also on the initiating stimulus and dying cells [11, 12]. Recent studies have shown that although chemotherapeutic agents are generally considered to be immunosuppressive, some chemotherapeutic agents can also activate the immune system. It has been reported that anthracycline can induce pyroptosis in tumor cells [11]. Pyroptosis is a lytic and inflammatory programmed cell death pathway mediated by GSDME, which is characterized by cell swelling and blowing of bullae from the plasma membrane, leading to cell death after rupture, and the formation of transmembrane pores to release DAMPs including HMGB1, ATP, calreticulin, and cytokines to activate the immune system [13, 18]. GSDME is a key factor in determining the mode of pyroptotic cell death [14, 17]. This study demonstrated that paclitaxel and doxorubicin, an anthracycline, induced pyroptosis in breast cancer cells with high GSDME expression, accompanied by GSDME cleavage, activation of caspase-3 and upstream caspase-9 proteins in the mitochondrial-mediated death pathway, LDH release and increased Annexin V+/PI+ cells. At the same time, the release of DAMPs, HMGB1 and ATP, were detected, suggesting that GSDME may mediate paclitaxel-induced pyroptosis in breast cancer cells through the caspase-9/caspase-3 pathway.

Monocyte/macrophage is part of the breast tumor microenvironment TILs [11]. It has two functional characteristics, one is to phagocytize granular antigens, and the other is to take up, process, and present antigens to T cells. It is an important antigen-presenting cell and plays an important role in inducing specific immune responses and neutralizing ICD [45]. M1 macrophages are classically activated macrophages, which can kill pathogens and tumor cells. The results of coculture of MDA-MB-231 cells and M1 macrophages showed that the phagocytosis of M1 macrophages was enhanced after paclitaxel administration, T cell activation assay showed that paclitaxel-treated MDA-MB-231 cells induced CD3+ T cells activation, as measured by an increase in IFN-γand IL-2 secretion, suggesting that paclitaxel may activate the anti-tumor immunity of macrophages through the pyroptosis pathway.

## Conclusion

To sum up, we used the ANN algorithm combined with the RF to develop the model for predicting the curative effect of neoadjuvant chemotherapy for breast cancer, which will help to identify which breast cancer patients benefit from paclitaxel and anthracycline-based neoadjuvant chemotherapy. Digital pathology, cytology, and molecular biology experiments further explored the mechanism of differential sensitivity to neoadjuvant chemotherapy. It was found that paclitaxel and anthracycline could induce pyroptosis of breast cancer cells and promote the release of DAMPs, suggesting that chemotherapeutic agents may work with TILs in tumor tissue to activate immune-mediated ICD and enhance anti-tumor immunity. Admittedly, the data set used in this study is retrospective, and prospective clinical validation is needed in the future. In addition, the interaction between pyroptosis and chemotherapeutic drugs and the specific mode of stimulating the immune response needs to be explored in animal and human samples. This study has a guiding role for the precise treatment of breast cancer in clinical practice and also provides new insights for research in the field of drug sensitivity.

### Supplementary Information

Below is the link to the electronic supplementary material.

Supplemental Figure 1 (A) Heat map of 351 differential genes in pCR samples and RD samples. (B) CD96 was a common gene in different models, and (C) PanglaoDB database analysis showed that CD96 was mainly expressed in T cells, NK cells, Gamma delta T cells, etc. (TIF 8987 KB)

Supplemental Figure 2 TILs evaluation and immunophenotyping in digital pathological slides of pretreatment breast tumor tissue from breast cancer patients (patients 1-18, P1-P18). (TIF 43638 KB)

Supplemental Figure 3 TILs evaluation and immunophenotyping in digital pathological slides of pretreatment breast tumor tissue from breast cancer patients (patients 19-36, P19-P36). (TIF 42991 KB)

Supplemental Figure 4 GSDME expression and drug-induced morphological changes in different breast cancer cell lines. (A) GSDME was highly expressed in MDA-MB-231 and MCF-7 cells, but not in T47D and EMT-6 cells. (B) Doxorubicin treatment of GSDME-overexpressing MDA-MB-231 and MCF-7 cells with high GSDME expression induced typical morphological changes of pyroptosis, but not in EMT-6 cells without GSDME expression at 24 h. (C) GSDME-negative T47D and EMT-6 cells did not show typical morphological features of pyroptosis such as cell swelling and plasma membrane bullae formation at 24 h after paclitaxel treatment. (TIF 27685 KB)

Supplemental Figure 5 Paclitaxel induces pyroptosis in GSDME-overexpressing 4T1 cells. (A) GSDME expression was examined in GSDME-overexpressing or wild-type 4T1 cells. (B) Typical pyroptosis-related morphological features in GSDME-overexpressing 4T1 cells after paclitaxel administration (50 nM). (C) The IL-1β level in cell culture supernatant after paclitaxel treatment (50 nM) were increased. 4T1-GSDME, GSDME-overexpressing 4T1 cells. *p < 0.05. (TIF 3817 KB)

Supplemental Table 1 Characteristics of datasets. (DOCX 12 KB)

## Data Availability

The original contributions presented in the study are included in the article/Supplementary Material. Further inquiries can be directed to the corresponding authors.
