# Supplementary figures and images for: GSDME-mediated pyroptosis promotes anti-tumor immunity of neoadjuvant chemotherapy in breast cancer
Source: Cancer Immunol Immunother. 2024 Jul 2;73(9):177. doi: 10.1007/s00262-024-03752-z (PMC11219631; doi:10.1007/s00262-024-03752-z)

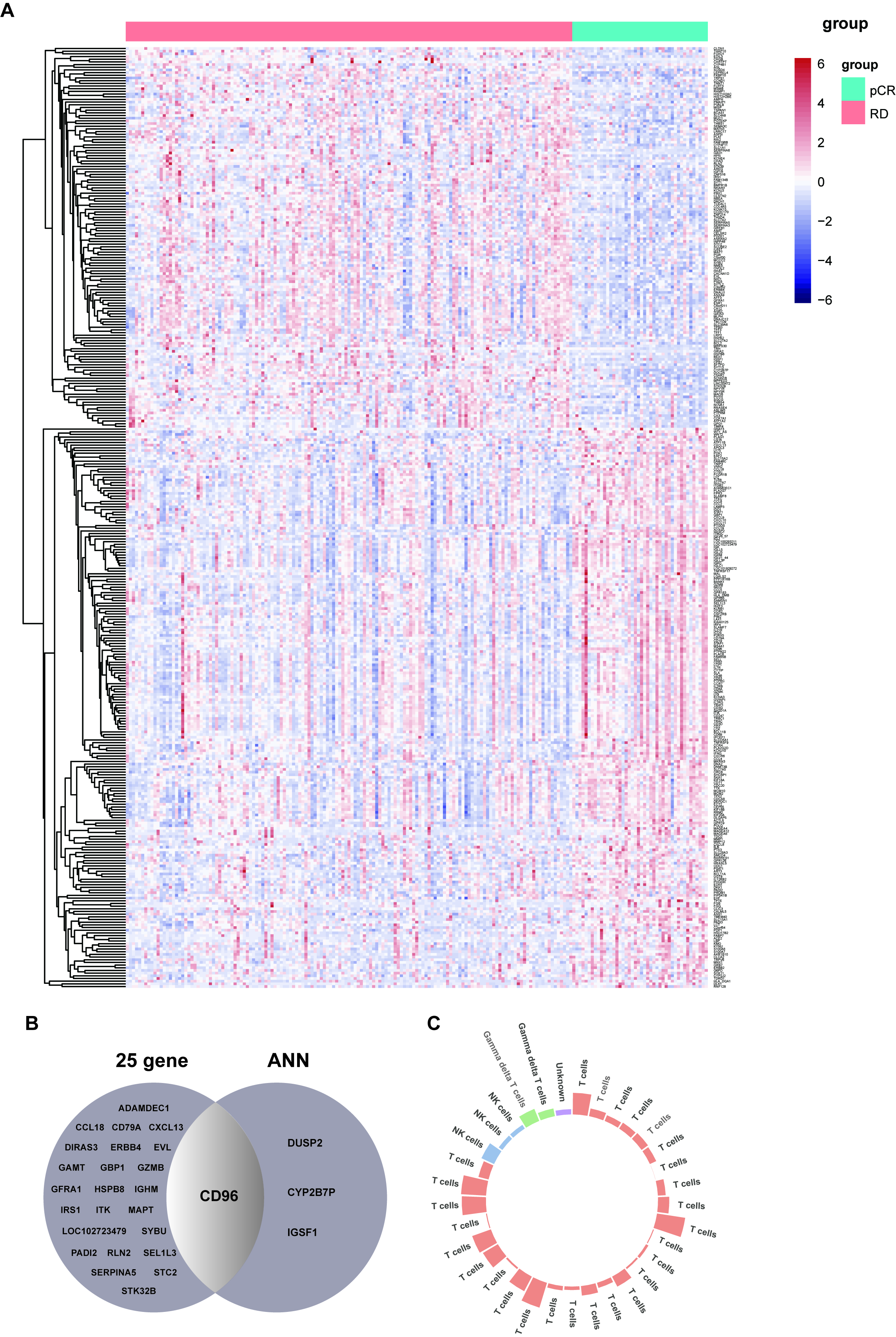

Supplement: Supplementary file 1 — Supplemental Figure 1 (A) Heat map of 351 differential genes in pCR samples and RD samples. (B) CD96 was a common gene in different models, and (C) PanglaoDB database analysis showed that CD96 was mainly expressed in T cells, NK cells, Gamma delta T cells, etc. (TIF 8987 KB) [file 262_2024_3752_MOESM1_ESM.tif]

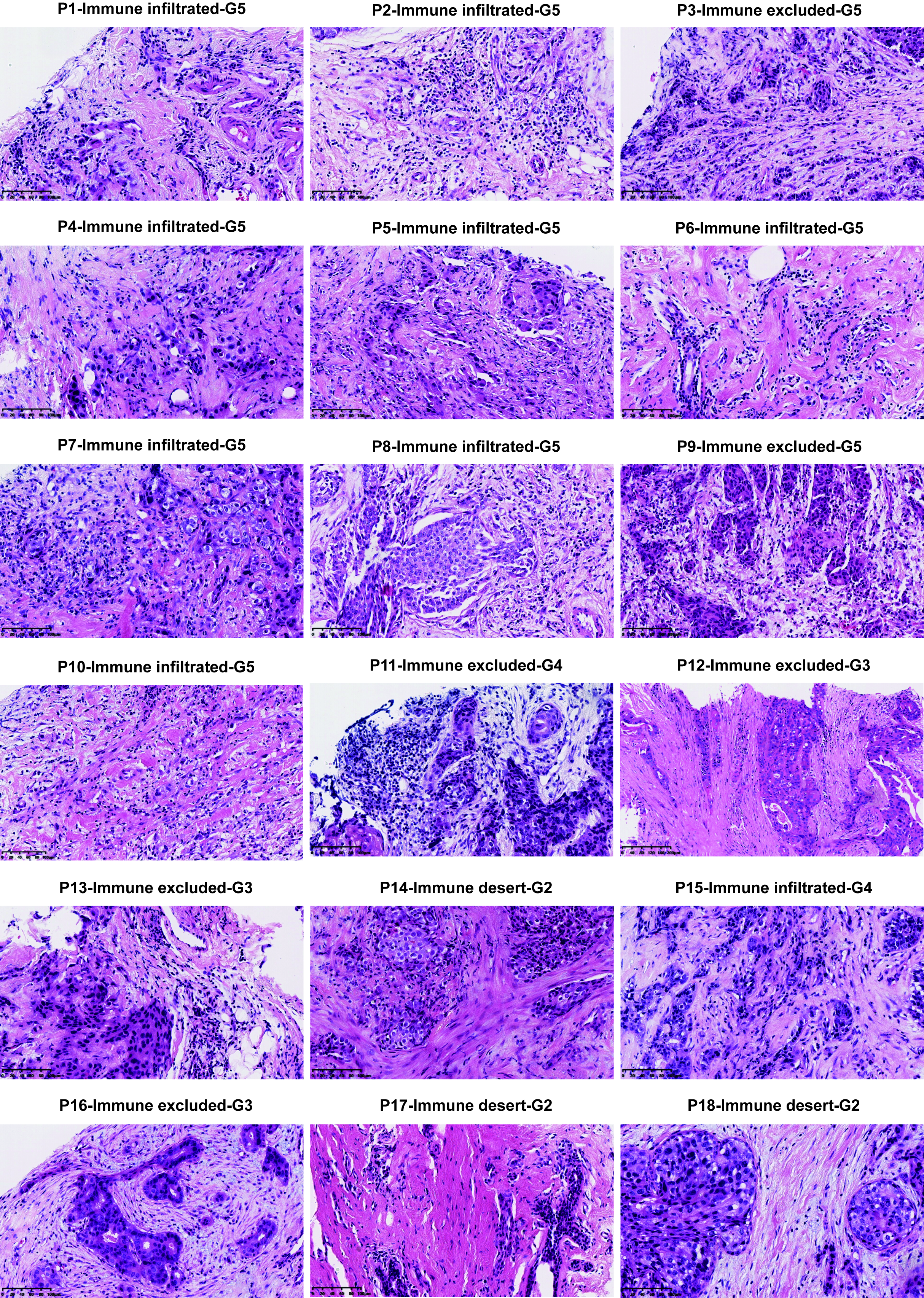

Supplement: Supplementary file 2 — Supplemental Figure 2 TILs evaluation and immunophenotyping in digital pathological slides of pretreatment breast tumor tissue from breast cancer patients (patients 1-18, P1-P18). (TIF 43638 KB) [file 262_2024_3752_MOESM2_ESM.tif]

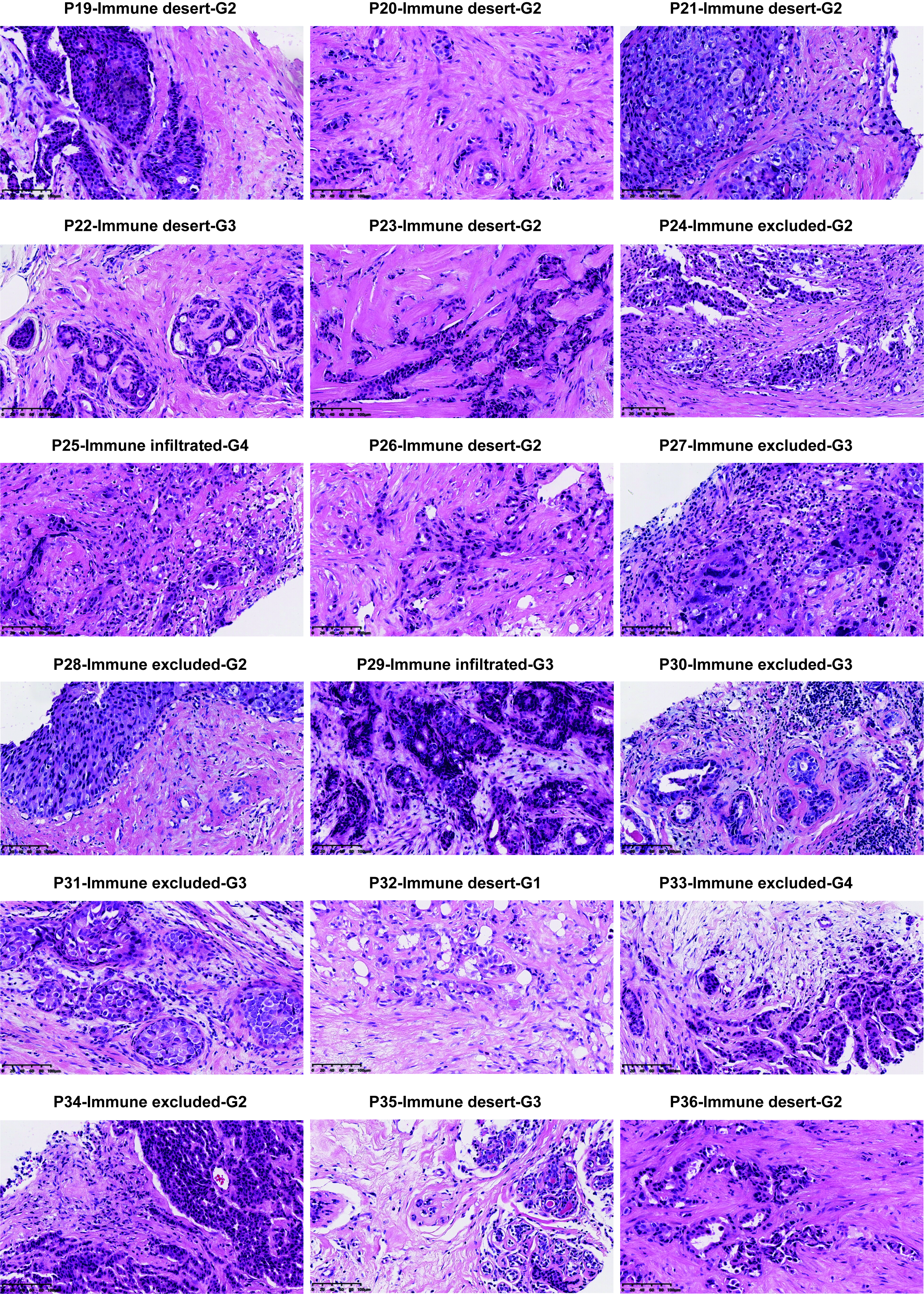

Supplement: Supplementary file 3 — Supplemental Figure 3 TILs evaluation and immunophenotyping in digital pathological slides of pretreatment breast tumor tissue from breast cancer patients (patients 19-36, P19-P36). (TIF 42991 KB) [file 262_2024_3752_MOESM3_ESM.tif]

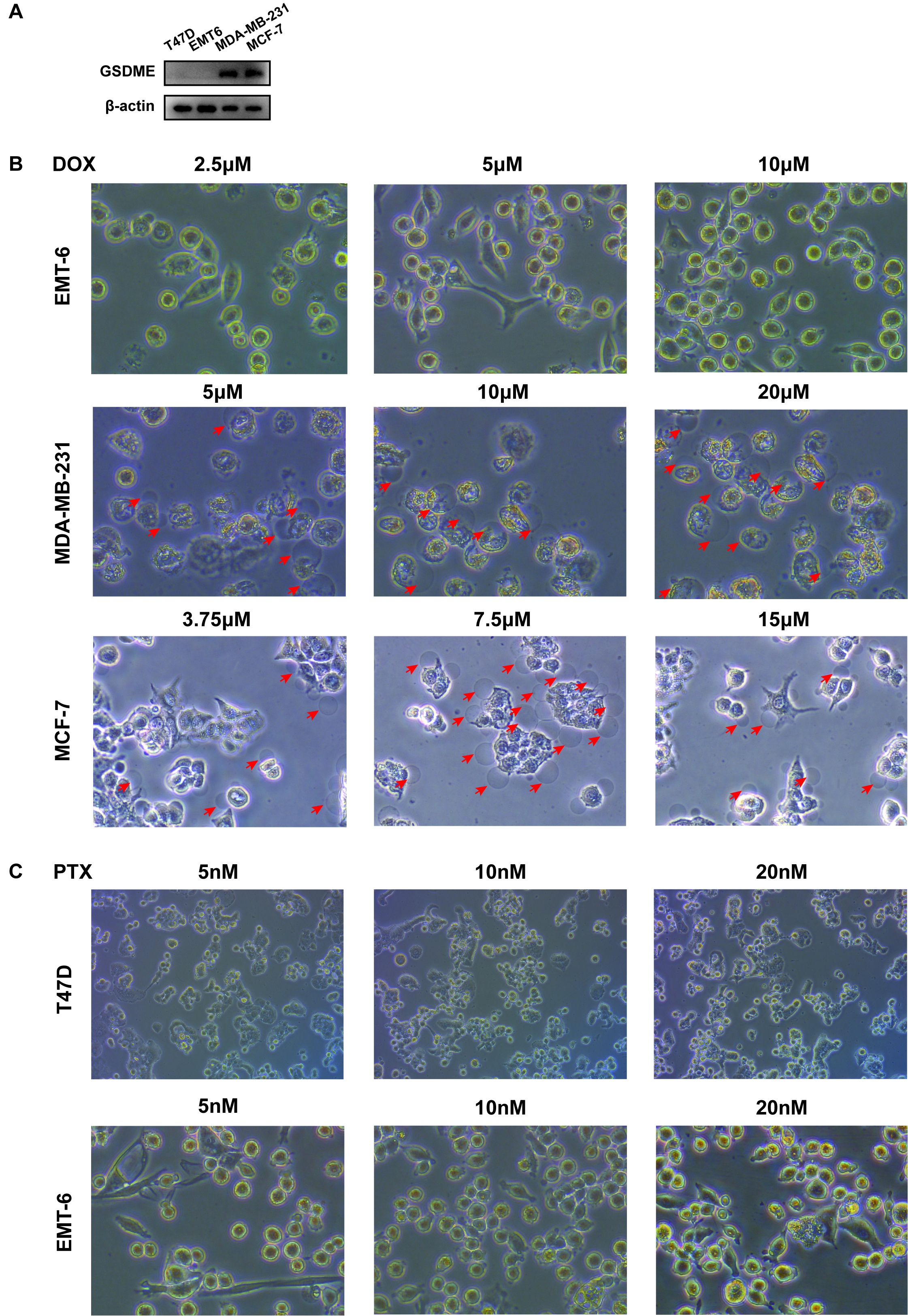

Supplement: Supplementary file 4 — Supplemental Figure 4 GSDME expression and drug-induced morphological changes in different breast cancer cell lines. (A) GSDME was highly expressed in MDA-MB-231 and MCF-7 cells, but not in T47D and EMT-6 cells. (B) Doxorubicin treatment of GSDME-overexpressing MDA-MB-231 and MCF-7 cells with high GSDME expression induced typical morphological changes of pyroptosis, but not in EMT-6 cells without GSDME expression at 24 h. (C) GSDME-negative T47D and EMT-6 cells did not show typical morphological features of pyroptosis such as cell swelling and plasma membrane bullae formation at 24 h after paclitaxel treatment. (TIF 27685 KB) [file 262_2024_3752_MOESM4_ESM.tif]

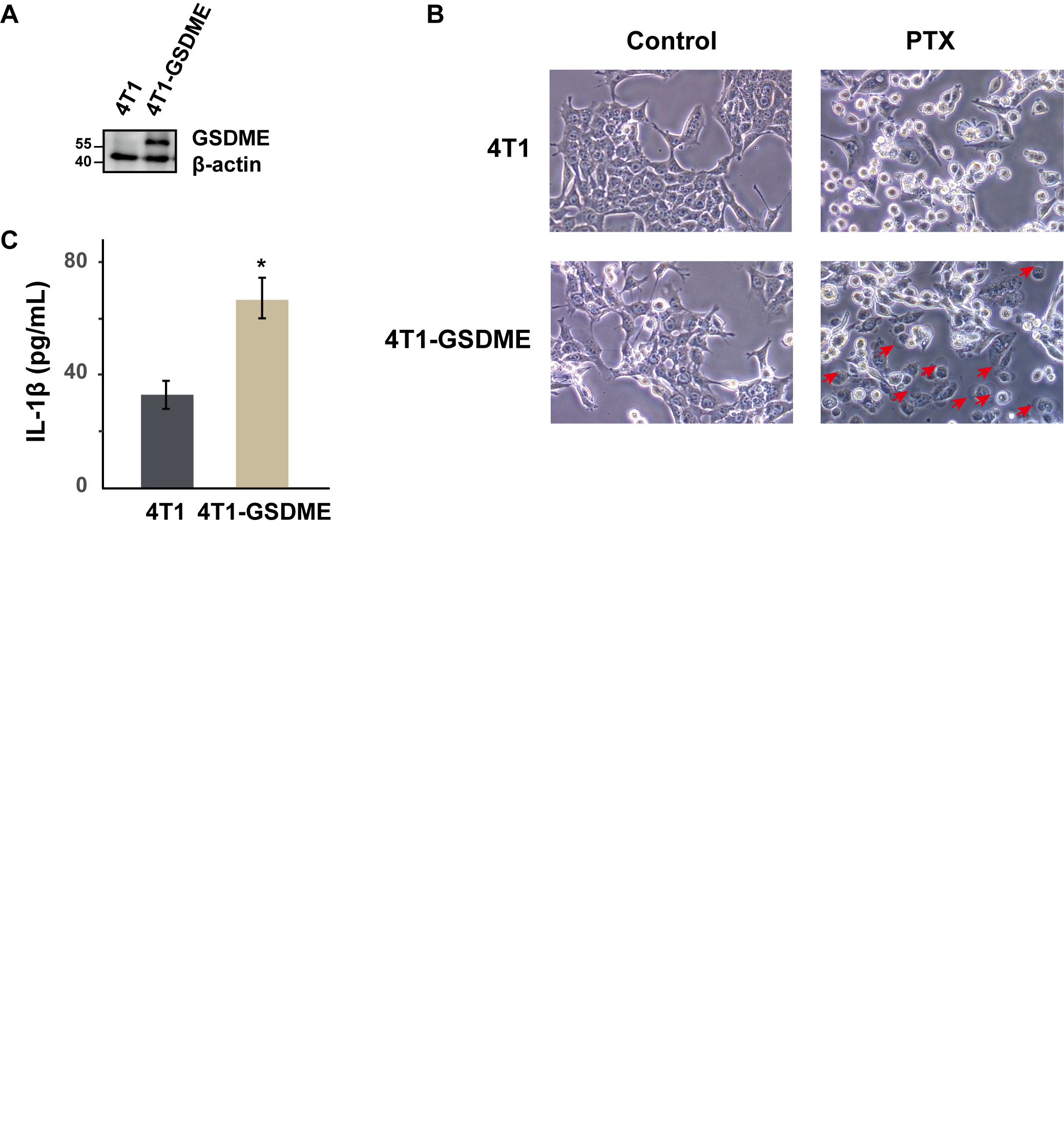

Supplement: Supplementary file 5 — Supplemental Figure 5 Paclitaxel induces pyroptosis in GSDME-overexpressing 4T1 cells. (A) GSDME expression was examined in GSDME-overexpressing or wild-type 4T1 cells. (B) Typical pyroptosis-related morphological features in GSDME-overexpressing 4T1 cells after paclitaxel administration (50 nM). (C) The IL-1β level in cell culture supernatant after paclitaxel treatment (50 nM) were increased. 4T1-GSDME, GSDME-overexpressing 4T1 cells. *p < 0.05. (TIF 3817 KB) [file 262_2024_3752_MOESM5_ESM.tif]
