# Supplementary material for: GSDME-mediated pyroptosis promotes anti-tumor immunity of neoadjuvant chemotherapy in breast cancer
Source: Cancer Immunol Immunother. 2024 Jul 2;73(9):177. doi: 10.1007/s00262-024-03752-z (PMC11219631; doi:10.1007/s00262-024-03752-z)
Supplement: Supplementary file 6 — Supplemental Table 1 Characteristics of datasets. (DOCX 12 KB) [file 262_2024_3752_MOESM6_ESM.docx]

**Supplemental Table 1 Characteristics of datasets**

| **Characteristic** | **GSE32646** | **GSE20271** | **GSE20194** | **GSE25055** | **GSE41998** |
| --- | --- | --- | --- | --- | --- |
| Platform | GPL570 | GPL96 | GPL96 | GPL96 | GPL571 |
| Samples | 115 | 74 | 207 | 227 | 121 |
| Molecular subtype | All | All | All | HER2- | All |
| Neoadjuvant therapy | T/FAC | T/FAC | T/FAC | T/FAC | T/AC |

T, paclitaxel; F, fluorouracil; A, anthracycline; C, cyclophosphamide.
